# Recommendation for a Standardised Method of Broth Microdilution Susceptibility Testing for Porcine *Bordetella bronchiseptica*

**DOI:** 10.1371/journal.pone.0123883

**Published:** 2015-04-24

**Authors:** Sandra Prüller, Cornelia Frömke, Heike Kaspar, Günter Klein, Lothar Kreienbrock, Corinna Kehrenberg

**Affiliations:** 1 Institute for Food Quality and Food Safety, University of Veterinary Medicine Hannover, Foundation, Bischofsholer Damm 15, D-30173, Hannover, Germany; 2 Department of Biometry, Epidemiology and Information Processing, WHO Collaborating Centre for Research and Training in Veterinary Public Health, University of Veterinary Medicine Hannover, Foundation, Hannover, Germany; 3 Federal Office of Consumer Protection and Food Safety, Berlin, Germany; Universidad Nacional de La Plata., ARGENTINA

## Abstract

The objective was to establish and standardise a broth microdilution susceptibility testing method for porcine *Bordetella *(*B*.) *bronchiseptica*. *B*. *bronchiseptica* isolates from different geographical regions and farms were genotyped by macrorestriction analysis and subsequent pulsed-field gel electrophoresis. One reference and one type strain plus two field isolates of *B*. *bronchiseptica* were chosen to analyse growth curves in four different media: cation-adjusted Mueller-Hinton broth (CAMHB) with and without 2% lysed horse blood, Brain-Heart-Infusion (BHI), and Caso broth. The growth rate of each test strain in each medium was determined by culture enumeration and the suitability of CAMHB was confirmed by comparative statistical analysis. Thereafter, reference and type strain and eight epidemiologically unrelated field isolates of *B*. *bronchiseptica* were used to test the suitability of a broth microdilution susceptibility testing method following CLSI-approved performance standards given in document VET01-A4. Susceptibility tests, using 20 antimicrobial agents, were performed in five replicates, and data were collected after 20 and 24 hours incubation and statistically analysed. Due to the low growth rate of *B*. *bronchiseptica*, an incubation time of 24 hours resulted in significantly more homogeneous minimum inhibitory concentrations after five replications compared to a 20-hour incubation. An interlaboratory comparison trial including susceptibility testing of 24 antimicrobial agents revealed a high mean level of reproducibility (97.9%) of the modified method. Hence, in a harmonization for broth microdilution susceptibility testing of *B*. *bronchiseptica*, an incubation time of 24 hours in CAMHB medium with an incubation temperature of 35°C and an inoculum concentration of approximately 5 x 10^5 ^cfu/ml was proposed.

## Introduction

*Bordetella bronchiseptica*, a strict aerobic, gram-negative coccobacillus belongs to the genus *Bordetella* within the *alcaligenaceae* family [1]. This pathogen is known to cause respiratory tract infections in mammals [2]. In infected pigs, the pathogen can lead to symptoms ranging from mild bronchitis to severe bronchopneumonia. It is also involved in atrophic rhinitis, associated with toxogenic strains of *Pasteurella multocida*, which can lead to severe distorsion and atrophy of the turbinate bones in the nasal terminus [3–5]. In dogs it causes tracheobronchitis and is one of the main pathogens of kennel cough [6]. Tracheobronchitis, conjunctivitis, rhinitis and pneumonia occurs in cats [7]. Respiratory tract infections due to *B*. *bronchiseptica* are also known in rabbits and guinea pigs [8].

Although primarily an animal pathogen, *B*. *bronchiseptica* may also cause pertussis-like respiratory symptoms in humans and is occasionally isolated from immunosuppressed hosts [9–11]. The pathogen is closely related to *Bordetella pertussis*, that causes whooping cough in humans, even though *B*. *bronchiseptica* does not harbor the same virulence factors [12, 13]. For systematic therapy of both human and animal patients and to reduce the chance of the selection of resistances against antimicrobial agents, increased knowledge about antimicrobial susceptibility of veterinary pathogens is required. A standardised method of susceptibility testing would provide more stable MIC data for treatment recommendations for veterinarians in the field.

However, currently there is a standardised protocol for non-fastidious, rapidly replicating bacteria available to perform broth microdilution susceptibility testing. Testing usually follows the recommendations given in CLSI-document VET01-A4, even though the applicability for *B*. *bronchiseptica* has not been demonstrated [14]. *B*. *bronchiseptica* is the least fastidious organism within the *Bordetella* genus, but due to its relatively slow growth compared to other rapidly replicating bacteria, it is necessary to prove if the same testing conditions apply [15]. Therefore, the aim of this study was to provide a basis for the harmonization of broth microdilution susceptibility testing for *B*. *bronchiseptica*.

## Materials and Methods

### Bacterial isolates and macrorestriction analysis

For this study, the reference strain DSM 10303 (Leibniz-Institute DSMZ, German Collection of Microorganism and Cellcultures, Braunschweig, Germany) and the type strain DSM 13414 (DSMZ) of *B*. *bronchiseptica* plus 17 porcine field isolates were used. The isolates were provided from diagnostic laboratories in Germany and originated from different farms and geographically distinct regions across Germany. They were isolated from both diseased and healthy swine collected between 2010 and 2012. *B*. *bronchiseptica* isolates were grown on Columbia sheep blood agar (Oxoid, Wesel, Germany) in ambient air at 35°C ± 1.0°C for 24 hours ± 2 hours. Species identification of the isolates was confirmed by a previously published *B*. *bronchiseptica* specific PCR which targets the flagellin *flaA* gene by amplification of a 237 bp fragment [11]. To investigate the clonality of the isolates, macrorestriction analysis was performed. For this, the XbaI digested fragments of *B*. *bronchiseptica* genomic DNA were separated in a CHEF DR II system (BioRad, Munich, Germany). The pulse time was increased from 7 to 20 s for 14 hours and from 30 to 50 s for 10 hours. The criterion of > 6 bands difference was used to define unrelated isolates, differences in 4–6 bands or 2–3 bands were defined as possibly related and closely related, respectively [16]. Based on the results of the macrorestriction analysis, two unrelated isolates (Bb5/12, Bb24/12) and both reference and type strains of *B*. *bronchiseptica* were chosen for the determination of growth curves. Isolates were collected in Germany in 2012 from diseased piglets. These four isolates plus six additional field isolates were used for susceptibility testing. The six field isolates were collected from different geographical regions and different farms in Germany 2010 to 2012 from diseased piglets and fattening pigs.

### Growth curves

Growth curves of the strains DSM 10303, DSM 13414, and field isolates Bb5/12 and Bb24/12 were generated over 48 hours by measuring the optical density OD_600_ on a UV-visible spectrophotometer (Varian Cary-100, Mulgrave Victoria, Australia), as well as by culture-based enumeration. For all four isolates an overnight culture was carried out in CAMHB (Oxoid, Wesel, Germany) at 35°C in ambient air. The overnight culture was adjusted to 0.5 McFarland standard and a 1:100 dilution was carried out in 0.9% saline. From this diluted bacterial suspension 100 μl was added to 100 ml of each medium to reach a start cfu/mL of approximately 10^3^ cfu/mL.

Isolates were cultured in cation-adjusted Mueller-Hinton broth (CAMHB), CAMHB plus 2% (v/v) lysed horse blood, Brain-Heart-Infusion (BHI) (Becton Dickinson, Heidelberg, Germany) and Caso broth (Sigma-Aldrich, Seelze, Germany), which are frequently used media for the cultivation of *Bordetella* spp. [17–19]. Incubation was performed at 35°C in ambient air. Sampling was performed every two hours during the first 26 hours and, thereafter every six hours. At each sampling time, starting at hour 0, a 10-fold serial dilution in 0.9% saline was done for every isolate, and four different dilutions were plated in duplicate on Columbia sheep blood agar. Enumeration plates were incubated at 35°C in ambient air. To calculate the average of colony forming units, two dilutions between 5 and approximately 200 cfu/mL were counted from the plated dilutions and the mean value of the two dilutions was determined. All experiments were performed three times independently.

### Susceptibility testing

The *B*. *bronchiseptica* reference and type strains (DSM 10303, DSM 13414) plus eight epidemiologically unrelated isolates or isolates differing in at least 3–6 bands were used to evaluate the suitability of broth microdilution susceptibility test conditions for *B*. *bronchiseptica*. Inoculum density (approximately 5 x 10^5^ cfu/mL), growth medium (CAMHB) and incubation conditions (35°C in ambient air) followed Clinical and Laboratory Standards Institute (CLSI, Wayne, PA, USA) guidelines for rapidly growing aerobic bacteria isolated from animals [14]. Isolates were investigated against a panel of 20 antibiotics/antibiotic combinations using a customised 50 μl/well volume microtiter plate (Sensititre, Trek Diagnostic Systems, East Grinstead, UK) layout. A broad range of antimicrobial agents (Table 1) was tested to prove the stability of the measured values across a broader spectrum, including several antimicrobial agents that are not currently licensed for food animals. In addition, some antimicrobial agents, for which a low susceptibility has been previously reported, e.g. cephalosporins, were included enabling assessment of homogeneity of MIC values in five replicates [1, 20]. MICs were determined visually for each isolate by the same person after 20 and 24 hours. Final inoculum concentration was verified by diluting the inoculum in a 10-fold serial dilution in 0.9% saline, plating the bacterial suspension on Columbia sheep blood agar and determining cfu on agar plates after incubation. An inoculum density of approximately 5 x 10^5^ cfu was targeted. For quality control purposes, the *Escherichia coli* strain ATCC 25922 was used, for which acceptable quality control ranges of MIC values for broth microdilution are available [14]. Tests were repeated independently five times.

**Table 1:** Tested antimicrobial agents and their ranges.

Antimicrobial agent	Ranges (μg/ml)
ampicillin	0.03–64
amoxicillin/clavulanic acid 2:1 ratio	0.03/0.015–64/32
ceftiofur	0.03–64
cefquinome	0.015–32
cefoperazone	0.06–32
cefotaxime	0.015–32
chloramphenicol	0.5–64
ciprofloxacin^1^	0.008–16
colistin	0.03–16
doxycycline	0.06–128
enrofloxacin	0.008–16
florfenicol	0.12–256
gentamicin	0.12–256
imipenem	0.015–32
marbofloxacin^1^	0.008–16
nalidixic acid	0.06–128
neomycin^1^	0.03–128
penicillin	0.015–32
spiramycin	0.06–128
streptomycin^1^	0.25–256
tiamulin	0.03–64
tetracycline	0.12–128
tilmicosin	0.06–64
trimethoprim/sulfamethoxazole (1:19 ratio)	0.015/0.3–32/608

^1^ these antimicrobial agents were included only in the interlaboratory comparison.

### Statistical analyses

The primary aim of this study was to compare the stability of MIC after 20 or 24 hours incubation time. Of secondary interest were the exploration of growth curves and the investigation of differences in MIC values depending on the incubation time.

To investigate whether the stability of the MIC values differed between the two incubation times, for each combination of isolate, antibiotic agent, and incubation time, MIC data consisting of five measurements was summarized to a dichotomous outcome. The outcome was set to 1 if all five measurements were equal and 0 otherwise. Stability of measurements was analyzed with a multi-factorial logistic regression model for mixed effects with dichotomous stability depending on incubation time, antibiotic agent and isolate (fixed effects) and identification of the stability measurement (random effect) [21]. Global *p*-values for all three factors were computed, and for the incubation time, the odds ratio with the corresponding two-sided 95%-confidence interval was estimated.

The growth of *B*. *bronchiseptica* isolates depending on the four media at a specific time point was computed with marginal confidence intervals based on an analysis of covariance (ANCOVA) for repeated measurements. For each individual strain, a model was computed consisting of the culture enumeration log-transformed to base 10 depending on time of measurement, medium, the interaction of time and medium, and the covariate baseline measurement. Furthermore, covariance among the time points was modelled with a first-order autoregressive structure [21]. Resulting confidence intervals of the interaction time by medium were examined for all medium-isolate combinations in three independent experiments.

Differences in MIC values depending on incubation time were investigated with an ANOVA with mixed effects modeling the log-transformed MIC values depending on the fixed factors of incubation time, antibiotic agent, and isolate, and sample identification as the random effect. Because the distribution of MIC values was skewed to the right and a two-fold dilution series was used, data was log-transformed to base 2. In addition to the global *p*-values for each factor, difference in incubation time means and the corresponding confidence interval on the logarithmic scale was estimated. After back-transformation, this estimate and the confidence interval corresponded to the ratio of geometric means of incubation times.

Except for the analysis of growth curves, interactions (e.g. odds ratios for incubation time dependent on antibiotic agent) were omitted in order to generalize usability. The type I error was set at 5% (two-sided). SAS System (Cary, NC, USA), Version 9.3., was used for all analyses [22].

### Interlaboratory comparison

An interlaboratory comparison, organized by the Federal Office of Consumer Protection and Food Safety (BVL, Berlin, Germany), was performed by a total of ten laboratories to determine the reproducibility of the method. All laboratories are experienced in conducting susceptibility testing of bacterial pathogens and they perform MICs routinely. *B*. *bronchiseptica* reference strain DSM10303, field isolate Bb5/12 and quality control strain *E*. *coli* ATCC25922 were sent to all participating laboratories together with microtiter plates (Sensititre, Trek Diagnostic Systems, East Grinstead, UK). For each replication, three microtiter plates with a total of 24 antimicrobial agents (Table 1) were used to obtain MIC data of broth microdilution susceptibility testing in CAMHB. An incubation temperature of 35°C and incubation time of 24 hours were chosen for the interlaboratory comparison. Cell densities and purity control were determined by colony count check immediately after inoculation and sub-culturing of strains, respectively, by each laboratory in each experimental replication. Testing in each laboratory took place within four weeks and was repeated independently three times. Analysis of MIC data was performed by determining the deviation of the modal value from MIC in log_2_-steps for each laboratory, bacterial strain and antimicrobial agent by the BVL, Berlin. Modal value plus minus one dilution step were chosen as a respectable determination.

## Results

### Clonality of *B*. *bronchiseptica* isolates and growth behavior

Although originating from different geographical regions in Germany, 11 of the 17 initially tested *B*. *bronchiseptica* isolates showed similar fragment patterns during macrorestriction analysis (Fig A in S1 File). Therefore, the two reference strains and eight field isolates (Bb24/12, Bb77/10, Bb5/12, Bb101/12, Bb41/12, Bb174/11, Bb164/11, Bb159/11) differing in at least 3 to >6 fragments were chosen for further comparison tests.

Growth curves of four isolates cultured in different media showed a difference in the results between optical density (OD_600_) and culture enumeration (cfu/mL). While the mean value of three replications of optical density measurement showed a beneficial growth in CAMHB + 2% lysed horse blood, the mean value of three replications of cfu/mL revealed an almost consistent growth in the four different media (Fig B–I in S1 File). However, results from culture-based enumeration demonstrated a slow but sufficient growth of *B*. *bronchiseptica* in CAMHB in comparison to other rapid replicating bacteria like *E*. *coli* or *Salmonella* Enteritidis [15, 23]. When considering single measuring points, statistical analysis revealed significant differences in growth for some isolate-media combinations, although all differences were less than one log step (Fig F–I in S1 File). The greatest difference was found for the DSM 10303 strain, where the difference CAMHB—CAMHB+HB after 14 hours incubation was -0.925 cfu/mL log10 (95%-confidence interval: -1.255; -0.595). However, after 22 to 24 hours, the growth of *B*. *bronchiseptica* reached the beginning of the stationary phase at approximately 10^9^ cfu/mL in all four media tested.

### Susceptibility testing

Overall, MIC values of the combination of ten isolates, twenty antimicrobial agents, two incubation times and five replicates resulting in 2,000 measurements were investigated (Table A in S2 File).

**Fig 1:**
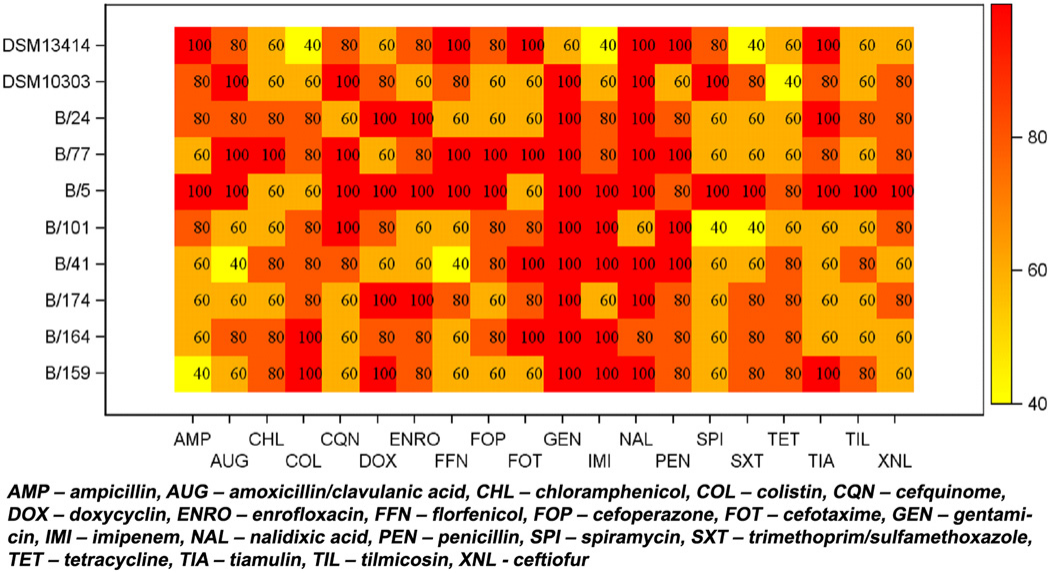
Heat map of the relative frequency [%] of MIC mode for each combination of antibiotic agent and bacterial strain (20 hours incubation time) demonstrated as heat map. A relative frequency of 100% demonstrates five equal measurements.

**Fig 2:**
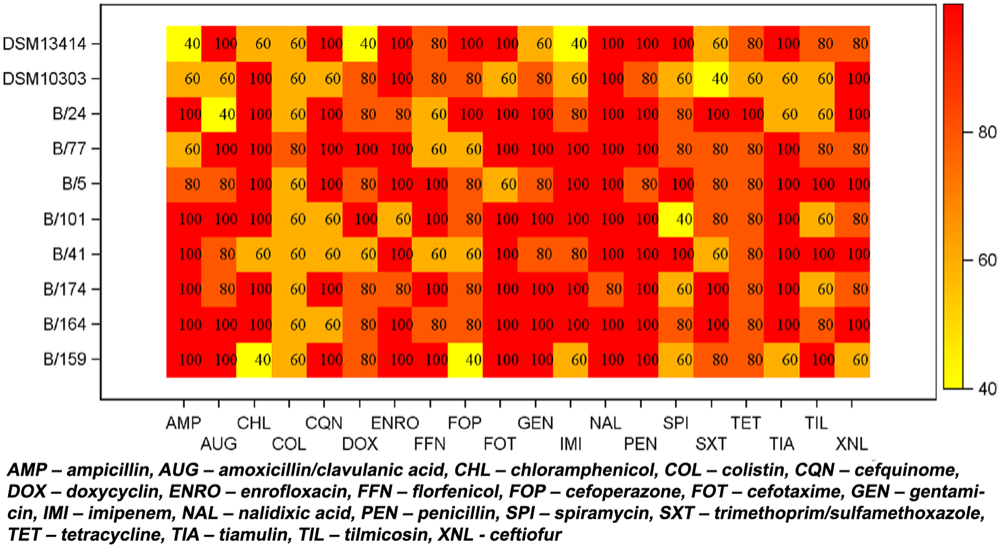
Heat map of the relative frequency [%] of MIC mode for each combination of antibiotic agent and bacterial strain (24 hours incubation time) demonstrated as heat map. A relative frequency of 100% demonstrates five equal measurements.

For statistical evaluation, each combination of five replicates was summarised to 0 (varying measurements) or 1 (stable results). The proportion of stable MIC values in the entire sample was 160/400 = 40%. After 24 hours of incubation, in 97 of 200 combinations (48.5%) of isolate and antibiotic agent the five MIC replicates were equal compared to 63 out of 200 (31.5%) after 20 hours of incubation. Detailed results of the relative frequencies of the MIC mode for each combination of antimicrobial agent and bacterial strain are shown in Figs 1 and 2 for 20 and 24 hours incubation time. In multi-factorial logistic regression analysis, the odds ratio for incubation time was 2.535 (95%-CI = [1.578; 4.071], *p* = 0.0001) stressing the higher stability of longer incubation. Differences in stability were also found among antibiotic agents (*p*≤0.0001) and isolates (*p* = 0.0293).

**Fig 3:**
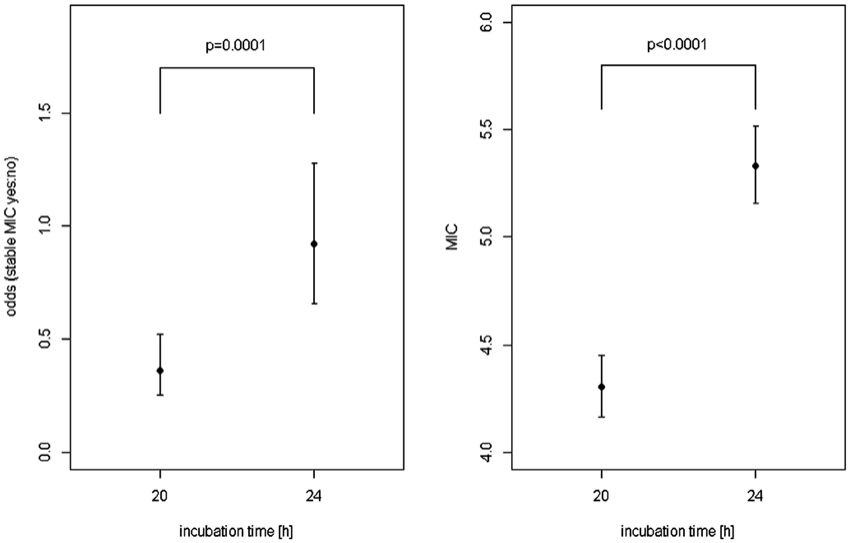
In the left graphic odds and the corresponding 95%-confidence intervals for stable MIC values split by incubation time are shown. Geometric means and corresponding 95%-confidence intervals for MIC values depending on incubation time are presented in the right graphic. Odds and geometric means are adjusted for bacteria strain and antibiotic agent.

Overall, increase in incubation time to 24 hours increased the geometric mean MIC value to 5.333 (9.351) compared to 4.305 (9.452) after 20 hours of incubation. In multi-factorial ANOVA, the ratio of geometric means was 1.239 (95%-CI = 1.181; 1.299, *p*≤0.0001) indicating an increase in MIC values with longer incubation (Fig 3). MIC values differed among the antibiotic agents (*p*≤0.0001) and isolates (*p*≤0.0001). Eight of 10 isolates showed more homogenous MICs after 24 hours than after the 20-hour incubation time recommended for rapidly growing bacteria. Only reference strain DSM 10303 and field isolate Bb5/12 showed more homogenous MICs after 20 hours incubation time. Ampicillin, amoxicillin/clavulanic acid, cefquinome, chloramphenicol, enrofloxacin, florfenicol, spiramycin tiamulin, tilmicosin and trimethoprim/sulfamethoxazole gave the most homogenous MIC values after 24 hours. In contrast, MICs for doxycycline and colistin were more homogenous after 20 hours. Three antimicrobial agents (nalidixic acid, gentamicin and cefotaxime) showed an equal distribution between 20 and 24 hours. Identical MIC values or more homogenous MICs after 24 hours were found in imipenem, tetracyclin, penicillin, ceftiofur and cefoperazone. All MIC values for the quality control strain *E*. *coli* ATCC25922 were within the acceptable ranges after 20 and 24 hours of incubation.

Possible violations of the ANOVA assumptions where investigated with a Q-Q-plot and a histogram of residuals. No considerable violations could be detected.

### Interlaboratory comparison

Reliable MIC data were collected by all laboratories participating in the interlaboratory comparison trial, whereby the data could be evaluated if the following criteria were met: 1. modus (entire number of most frequent determined MICs) and value of participant was equal or differed by plus minus one dilution step for the combination of bacterial strain and antimicrobial agent; 2. MIC values of reference strain were in context with reference range for each antimicrobial agent; 3. positive growth control in both wells of microtiterplates; 4. no contamination was verified in bacterial inoculum and 5. inoculum density exhibited a CFU/mL between 3 x10^5^ and 5 x 10^5^ cfu.

A conformance of 96.7% was accomplished for the field isolate Bb5/12, 98.1% for the type strain DSM 10303 and 98.9% for *E*. *coli* ATCC25922 by all laboratories with a mean of 97.9%. One laboratory showed a mode of conformance of 94%, two laboratories reached 100% and three greater than 99%. Four laboratories ranged between 95.8% and 96.9% for all bacterial species. MICs of each antimicrobial agent ranged between 94.7% and 98.8% conformance, MICs of colistin (90%) and ceftiofur (91.1%) reached a relatively lower conformity whereas ampicillin, cefoperazone, cefotaxime, ciprofloxacin, enrofloxacin, gentamicin, imipeneme, nalidixic acid and tilmicosin attained 100% conformity. The mean value of the interlaboratory reproducibility for all antimicrobial agents was 97.9%.

## Discussion

Within the genus *Bordetella*, the species *B*. *bronchiseptica* is less fastidious than the human bacterial pathogen *B*. *pertussis* [15]. Nevertheless, it is important to examine if testing conditions given in CLSI-document VET01-A4 for rapidly growing bacteria isolated from animals apply to porcine *B*. *bronchiseptica*. For this, growth curves were performed at 35°C in four different media, which are frequently used for a cultivation of *Bordetella* spp. Surprisingly, results of growth curves from optical density readings and culture enumeration differed with OD_600_ measurements falsely hinting at beneficial growth of *B*. *bronchiseptica* in CAMHB + 2% lysed horse blood. These results pointed out that OD measurement is not adequate for growth determinations of *B*. *bronchiseptica* for use with blood-containing media. The tendency of blood to darken upon exposure to incubation temperatures may be a reason for this observation. Differences in growth parameters derived from OD readings and colony counts have been reported previously (e.g. for *Listeria monocytogenes*) and should be considered when analysing bacterial growth based on OD data [24, 25].

As culture enumeration detected an almost consistent growth in all media, CAMHB was chosen for susceptibility testing. CAMHB is a suitable medium for this purpose and is recommended in the CLSI-guidelines for non-fastidious organisms because of its fairly good batch-to-batch reproducibility for broth dilution susceptibility testing and a low amount of inhibitors for testing trimethoprim, sulphonamides or tetracyclins [14].

Nevertheless, the lower growth rate of *B*. *bronchiseptica* compared to other rapidly replicating bacteria indicated that the incubation time should be increased. This was underlined by statistical analysis of repeated susceptibility testings of 10 *B*. *bronchiseptica* isolates, showing a comparative advantage of 24 hours incubation over 20 hours incubation. For these testings, as many unrelated *B*. *bronchiseptica* as possible were chosen, even though results from macrorestiction analysis strongly suggested a clonal relationship between some of the isolates, as previously reported for *B*. *bronchiseptica* isolates from Germany or Korea [8, 26]. It cannot be excluded that an incubation time longer than 24 hours would lead to more stable results; however, when considering that the beginning of the stationary phase of growth curves was reached after 24 hours, extension of the incubation time would be more in line with the testing of rapidly growing bacteria like *Salmonella* and *E*. *coli*. For an incubation time longer than 24 hours, stability of the antimicrobial agents should be verified by using QC-strains with their established MICs and, furthermore, this may be inappropriate for routine diagnostic applications.

Interlaboratory comparisons of the method described here delivered reliable results for broth microdilution susceptibility testing of *B*. *bronchiseptica* in CAMHB with 24 hours incubation time and showed a high level of reproducibility. Only minor variances were seen between the different laboratories. This outcome was in accordance to the statistical analysis of the stability of MIC values between 20 and 24 hours incubation time, which showed more homogenous results after 24 hours incubation time. That a 24 hour incubation time is comparatively advantageous for more homogenous MICs is in accordance with recommendations for fastidious or special problem veterinary pathogens, such as *Campylobacter* spp. or *Histophilus somni*, for which modified incubation times are required [14].

However, the increase in incubation time to 24 hours may lead to minor differences in isolate classification according to the veterinary porcine respiratory tract specific *B*. *bronchiseptica* breakpoints. Veterinary specific *B*. *bronchiseptica* breakpoints are only available for ampicillin, florfenicol and tulathromycin (not tested) [14]. These breakpoints were created by using the already existing method of the CLSI guidelines in document VET01-A4 for rapidly replicating pathogens of bacteria isolated from animals. Increasing the incubation time to 24 hours would not lead to a different classification of all 10 porcine *B*. *bronchiseptica* isolates for ampicillin. For florfenicol, there would be no change from susceptible or intermediate to resistant in all 10 porcine isolates. However, a change from susceptible to intermediate is possible for six of the 10 porcine isolates. Whether changing the incubation time to 24 hours would lead to changes in MIC_50_ and MIC_90_ values for antimicrobials without established breakpoints remains to be elucidated and should be investigated by testing a larger number of *B*. *bronchiseptica* isolates.

Even though only one isolate from a dog (type strain DSM 13414) was tested, the growth curves of the type strain DSM 13414 showed the same low growth rate and reached the stationary phase after 24 hours. However, to confirm the suitability of the method for isolates from companion animals, further studies are needed for the testing of isolates from other animal origins than swine.

## Conclusion

CAMHB is a suitable medium to use for broth microdilution susceptibility testing of porcine *B*. *bronchiseptica* while still using the recommended inoculum density of approximately 5 x 10^5^ cfu/mL and the incubation temperature of 35°C. An incubation time of 24 hours of the microtiter plates could be useful to produce the most reliable and consistent results.

## Supporting Information

S1 File **Fig. A**. Results of macrorestriction analysis from 17 *B*. *bronchiseptica* isolates using *Salmonella* Typhimurium LT2 marker and restriction-endonuclease XbaI. **Fig. B**. Optical density (OD) measurement of *B*. *bronchiseptica* reference strain DSM 10303 in four media; mean of three independent experiments. **Fig. C**. Optical density (OD) of *B*. *bronchiseptica* type strain DSM 13414 in four media, mean of three independent experiments. **Fig. D**. Optical density (OD) of *B*. *bronchiseptica* field isolate Bb5/12 in four media, mean of three independent experiments. **Fig. E**. Optical density (OD) of *B*. *bronchiseptica* field isolate Bb24/12 in four media, mean of three independent experiments. **Fig. F**. Number (Log cfu/ml) of bacteria (*B*. *bronchiseptica* reference strain DSM 10303) in four media; mean of three independent experiments. **Fig. G**. Number (Log cfu/ml) of bacteria (*B*. *bronchiseptica* type strain DSM 13414) in four media; mean of three independent experiments. **Fig. H**. Number (Log cfu/ml) of bacteria (*B*. *bronchiseptica* field isolate Bb5/12) in four media; mean of three independent experiments. **Fig. I**. Number (Log cfu/ml) of bacteria (*B*. *bronchiseptica* field isolate Bb24/12) in four media; mean of three independent experiments.
(DOCX)

S2 File **Table A**. MIC values of 10 *B*. *bronchiseptica* isolates, 20 antimicrobial agents and two incubations times in five replicates.
(XLSX)
