# Supplementary material for: Recommendation for a Standardised Method of Broth Microdilution Susceptibility Testing for Porcine Bordetella bronchiseptica
Source: PLoS One. 2015 Apr 24;10(4):e0123883. doi: 10.1371/journal.pone.0123883 (PMC4409320; doi:10.1371/journal.pone.0123883)
Supplement: S1 File — Fig. A. Results of macrorestriction analysis from 17 B. bronchiseptica isolates using Salmonella Typhimurium LT2 marker and restriction-endonuclease XbaI. Fig. B. Optical density (OD) measurement of B. bronchiseptica reference strain DSM 10303 in four media; mean of three independent experiments. Fig. C. Optical density (OD) of B. bronchiseptica type strain DSM 13414 in four media, mean of three independent experiments. Fig. D. Optical density (OD) of B. bronchiseptica field isolate Bb5/12 in four media, mean of three independent experiments. Fig. E. Optical density (OD) of B. bronchiseptica field isolate Bb24/12 in four media, mean of three independent experiments. Fig. F. Number (Log cfu/ml) of bacteria (B. bronchiseptica reference strain DSM 10303) in four media; mean of three independent experiments. Fig. G. Number (Log cfu/ml) of bacteria (B. bronchiseptica type strain DSM 13414) in four media; mean of three independent experiments. Fig. H. Number (Log cfu/ml) of bacteria (B. bronchiseptica field isolate Bb5/12) in four media; mean of three independent experiments. Fig. I. Number (Log cfu/ml) of bacteria (B. bronchiseptica field isolate Bb24/12) in four media; mean of three independent experiments. (DOCX) [file pone.0123883.s001.docx]

**Supporting Information S1 File**

**Fig. A**

1 2 3 4 5 6 7 8 9 10 11 12 13 14 15 16 17


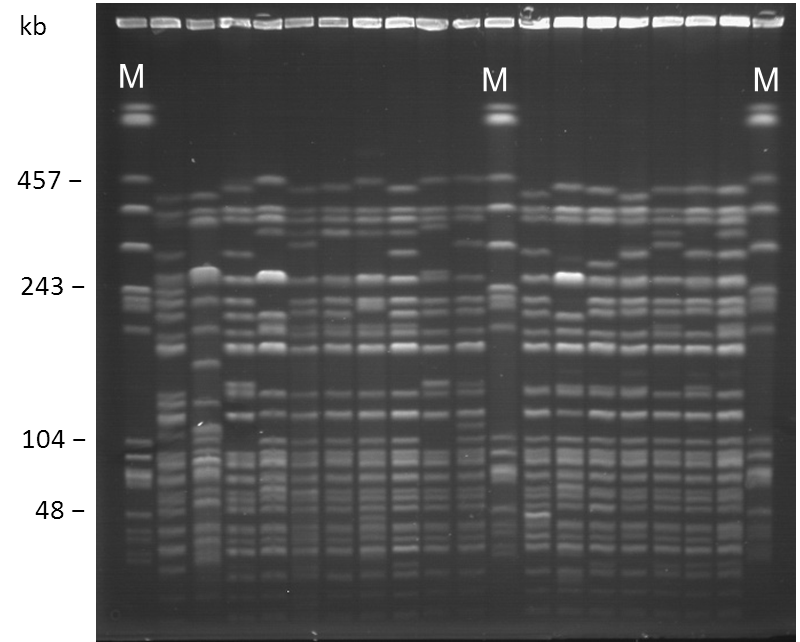


Lane 1: DSM 10303; lane 2: DSM 13414; lane 3: Bb24/12; lane 4: Bb5/12; lane 5: Bb77/10; lane 6: Bb41/12; lane 7: Bb174/11; lane 8: Bb159/11; lane 9: Bb164/11; lane 10: Bb101/12; lane 11: Bb140/12; lane 12: Bb157/11; lane13: Bb70/12; lane 14 Bb55/12; lane 15: Bb92/11; lane 16: Bb15/11; lane 17: Bb17/12; lanes M: size marker

**Fig. B**

CAMHB – cation-adjusted Mueller-Hinton Broth

CAMHB + 2% HB – cation-adjusted Mueller-Hinton Broth + 2 % horseblood

BHI – brain heart infusion

Caso – caso broth

**Fig. C**

CAMHB – cation-adjusted Mueller-Hinton Broth

CAMHB + 2% HB – cation-adjusted Mueller-Hinton Broth + 2 % horseblood

BHI – brain heart infusion

Caso – caso broth

**Fig. D**

CAMHB – cation-adjusted Mueller-Hinton Broth

CAMHB + 2% HB – cation-adjusted Mueller-Hinton Broth + 2 % horseblood

BHI – brain heart infusion

Caso – caso broth

**Fig. E**

CAMHB – cation-adjusted Mueller-Hinton Broth

CAMHB + 2% HB – cation-adjusted Mueller-Hinton Broth + 2 % horseblood

BHI – brain heart infusion

Caso – caso broth

**Fig. F**

CAMHB – cation-adjusted Mueller-Hinton Broth

CAMHB + 2% HB – cation-adjusted Mueller-Hinton Broth + 2 % horseblood

BHI – brain heart infusion

Caso – caso broth

**Fig. G**

CAMHB – cation-adjusted Mueller-Hinton Broth

CAMHB + 2% HB – cation-adjusted Mueller-Hinton Broth + 2 % horseblood

BHI – brain heart infusion

Caso – caso broth

**Fig. H**

CAMHB – cation-adjusted Mueller-Hinton Broth

CAMHB + 2% HB – cation-adjusted Mueller-Hinton Broth + 2 % horseblood

BHI – brain heart infusion

Caso – caso broth

**Fig. I**

CAMHB – cation-adjusted Mueller-Hinton Broth

CAMHB + 2% HB – cation-adjusted Mueller-Hinton Broth + 2 % horseblood

BHI – brain heart infusion

Caso – caso broth
